# Case report: Better late than never, but sooner is better: switch from CSII to sulfonylureas in two patients with neonatal diabetes due to KCNJ11 variants

**DOI:** 10.3389/fendo.2023.1143736

**Published:** 2023-05-11

**Authors:** Valentina Mancioppi, Erica Pozzi, Sara Zanetta, Anna Missineo, Silvia Savastio, Fabrizio Barbetti, Simona Mellone, Mara Giordano, Ivana Rabbone

**Affiliations:** ^1^ Division of Pediatrics, Department of Health Sciences, University of Piemonte Orientale, Novara, Italy; ^2^ Neonatal and Pediatric Intensive Care Unit, Azienda Ospedaliero Universitaria Maggiore della Carità, Novara, Italy; ^3^ Monogenic Diabetes Clinic, Bambino Gesù Children’s Hospital, Scientific Institute for Research, Hospitalization and Health Care (IRCCS), Rome, Italy; ^4^ Laboratory of Genetics, Struttura Complessa a Direzione Universitaria (SCDU) Biochimica Clinica, Azienda Ospedaliero Universitaria Maggiore della Carità, Novara, Italy; ^5^ Department of Health Sciences, University of Piemonte Orientale, Novara, Italy

**Keywords:** neonatal diabetes mellitus (NDM), monogenic diabetes, KCNJ11, continuous subcutaneous insulin infusion (CSII), glibenclamide, hyperglycemia

## Abstract

Neonatal diabetes mellitus (NDM) is a rare genetic disease characterized by severe hyperglycemia requiring insulin therapy with onset mostly within the first 6 months and rarely between 6-12 months of age. The disease can be classified into transient (TNDM) or permanent neonatal diabetes mellitus (PNDM), or it can be a component of a syndrome. The most frequent genetic causes are abnormalities of the 6q24 chromosomal region and mutations of the ABCC8 or KCNJ11 genes coding for the pancreatic beta cell’s potassium channel (KATP). After the acute phase, patients with ABCC8 or KCNJ11 mutations treated with insulin therapy can switch to hypoglycemic sulfonylureas (SU). These drugs close the KATP channel binding the SUR1 subunit of the potassium channel and restoring insulin secretion after a meal. The timing of this switch can be different and could affect long-term complications. We describe the different management and clinical outcome over the time of two male patients with NDM due to KCNJ11 pathogenetic variants. In both cases, continuous subcutaneous insulin infusion pumps (CSII) were used to switch therapy from insulin to SU, but at different times after the onset. The two patients kept adequate metabolic control after the introduction of glibenclamide; during the treatment, insulin secretion was evaluated with c-peptide, fructosamine, and glycated hemoglobin (HbA1c), which were within the normal range. In neonates or infants with diabetes mellitus, genetic testing is an indispensable diagnostic tool and KCNJ11 variants should be considered. A trial of oral glibenclamide must be considered, switching from insulin, the first line of NDM treatment. This therapy can improve neurological and neuropsychological outcomes, in particular in the case of earlier treatment initiation. A new modified protocol with glibenclamide administered several times daily according to continuous glucose monitoring profile indications, was used. Patients treated with glibenclamide maintain good metabolic control and prevent hypoglycemia, neurological damage, and apoptosis of beta cells during long‐term administration.

## Introduction

Neonatal diabetes mellitus (NDM) is a genetic disease characterized by severe hyperglycemia requiring insulin therapy, occurring mostly within the first six months of life, and rarely between six and twelve months of age (1–5). The incidence of NDM is estimated to be 1:20.000-500.000 live births (6). In Italy, the incidence is reported to be 1:90.000 live birth (7).

Clinically, the disease can be classified into transient neonatal diabetes mellitus (TNDM), permanent neonatal diabetes mellitus (PNDM), or it can be a component of a syndrome. TNDM accounts for 50% of all NDM cases, with diabetes resolving within 18 months of age, with a median resolution age of 14-18 weeks (3, 4, 8). Unfortunately, TNDM can relapse at the time of puberty in about 50% of patients (9). In the remaining cases, known as PNDM, children will require treatment during their life to control their glycemic profile.

More than 30 genes have been associated with NDM so far (10, 11). The underlying mechanisms involved in the development of NDM are pancreas malformation or abnormal function of the β cells responsible for insulin synthesis or secretion (3, 12). The first subgroup involves genes related to the pancreas development at various stages in the morphogenesis, causing pancreatic hypoplasia or aplasia; these cases typically present both exocrine pancreas deficiency and other congenital malformations, especially in the digestive and cardiac systems (13). About 80% of children with NDM have genetic mutations affecting the pancreatic β cells, mainly abnormalities of chromosome region 6q24 and pathogenic variants of the ABCC8 or KCNJ11 genes coding for the ATP-dependent potassium (KATP) channel. The KATP channel can regulate insulin secretion by linking glucose metabolism and ATP production: higher glucose level within the β cell increases the intracellular ATP concentration, with the subsequent closing of the KATP channel leading to cell membrane depolarization that triggers insulin secretion (10, 13). Activating mutations in ABCC8 or KCNJ11 prevent the channel closure, the insulin cannot be released within the bloodstream, and the blood glucose level keeps increasing till the development of NDM symptoms (14–16). KATP channels are expressed not only in the pancreatic β cells but also in the neurons and muscle cells so that patients can present a wide range of neurocognitive disabilities such as language disorders during development, sleep and hyperactivity disorders, and, in some cases, children may also be affected by DEND (developmental delay, epilepsy, and NDM) syndrome (3, 17, 18).

NDM diagnosis can be difficult because of the different causes of hyperglycemia in neonates, especially in the preterm population or low birth weight infants, such as sepsis, increased counter-regulatory hormones due to stress, parenteral glucose administration and medications such as steroids and beta-adrenergic agents have to be considered as differential diagnosis (19). Besides, TNDM and PNDM have the same clinical presentation, so there is a significant overlap between these two types at the onset of neonatal diabetes, and the clinical course of the disease cannot be predicted at the time of diagnosis.

The management of patients with NDM can be complicated, and continuous subcutaneous insulin infusion pumps (CSII) are considered the gold standard of insulin therapy, permitting the administration of very small insulin doses and avoiding severe hypoglycemic episodes due to the unpredictable feeding patterns of the neonates (8, 20). After the acute phase, patients with ABCC8 or KCNJ11 mutations treated with insulin therapy can switch to off-label hypoglycemic sulfonylureas (SU) (3, 13, 21). These drugs close the KATP channel, binding the SUR1 subunit of the potassium channel and restoring insulin secretion after a meal (22, 23). In about 90-95% of cases, the mutated channels are sensitive to sulfonylureas (24–27). SU can also improve neurological abnormalities in these patients, and early treatment initiation was associated with greater benefits, especially for epilepsy, muscular tone, and motor function (13, 21, 28).

Here we describe the different management and clinical outcome over the time of two male patients with NDM due to KCNJ11 pathogenetic variants.

## Case series presentation

### Infant 1

An 18-year-old boy was referred to our Unit from another Diabetes Centre for re-evaluation and follow-up prosecution. When the child was two months old, he was admitted to the hospital for fever without localizing signs and symptoms; the diagnostic work-out revealed hyperglycemia without diabetic ketoacidosis (DKA). No family history of diabetes was reported. Insulin therapy was immediately initiated, first intravenously, then with multiple daily injections (MDI) associated with self-blood glucose monitoring (SBGM). In the absence of specific autoantibodies for type 1 diabetes mellitus (anti-insulin, anti-glutamic decarboxylase, anti-pancreatic islet cell, and anti-tyrosine phosphatase were negative), NDM was clinically suspected but not genetically confirmed (no known mutations found at the genetic analysis). At nine months of age, in consideration of the small doses of insulin requirement and the difficulty to manage the high glycemic variability, he was switched to CSII treatment. Furthermore, language delay was evident at four to five years of age, although motor development was normal. At the age of 5 years old, despite the absence of a genetic diagnosis, with parents’ informed consent, the boy was gradually weaned from insulin therapy and switched to off-label glibenclamide (starting dose 0,15 mg/kg/day divided in 3-4 administrations) and subcutaneous insulin therapy was discontinued, with good results. During the follow-up, the patient underwent brain magnetic resonance imaging (MRI) that identified two symmetrical areas of altered signal compatible with gliosis of the cerebellar parenchyma with dysmetabolic genesis. After SU therapy was started, an improvement in language skills and metabolic response (HbA1c below 7%) was observed, but during the follow-up period, the boy started therapy with valproic acid due to a worsening cognitive disorder. No other complications or side effects were observed. At our first evaluation, when the patient was 18 years old, he was in good clinical condition, with optimal glycemic control (Hba1c 6.4%) and a good neurological outcome, with only a slight cognitive disorder. He was being treated with glibenclamide (0.1 mg/kg/day divided into 4 administrations, before meals, and at bedtime to avoid glycemic peaks during the night titrated thanks to SBGM. In the suspicion of PNDM, based on the clinical history and the good response to SU, a targeted NGS panel for monogenic diabetes was performed, with the detection of a *de novo* heterozygous nonsynonymous pathogenic mutation in *KCNJ11* (c.601C>T, p.Arg201Cys). After the genetic diagnosis, an attempt to shift therapy from glibenclamide to gliclazide (starting dose 0,24 mg/kg/day till a maximum of 0.95 mg/kg/day) was made, without improvement in the glycemic control and glibenclamide therapy was, therefore, restarted the following month. Currently, the boy is pursuing the same treatment (glibenclamide 0.15 mg/kg/day divided into 4 administrations) with optimal glycemic control (HbA1c 6.6%), always monitored by SBGM without continuous glucose monitoring (CGM).

### Infant 2

**Figure 1:**
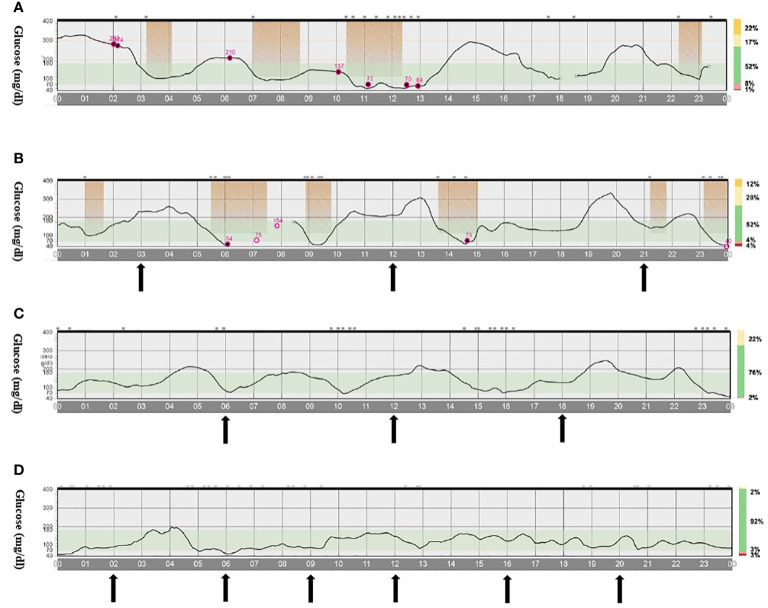
CGM profile of infant 2 during treatment with insulin and/or glibenclamide. **(A)**. Only CSII therapy (both basal and bolus delivered by CSII). **(B)**. CSII (only basal insulin) and glibenclamide therapy divided into three meals. **(C)**. Only glibenclamide therapy divided in three meals. **(D)**. Only glibenclamide therapy divided into six meals. The arrows indicate the administration of glibenclamide during meals. Abbreviations: CSII, continuous subcutaneous insulin infusion pumps.

**Figure 2:**
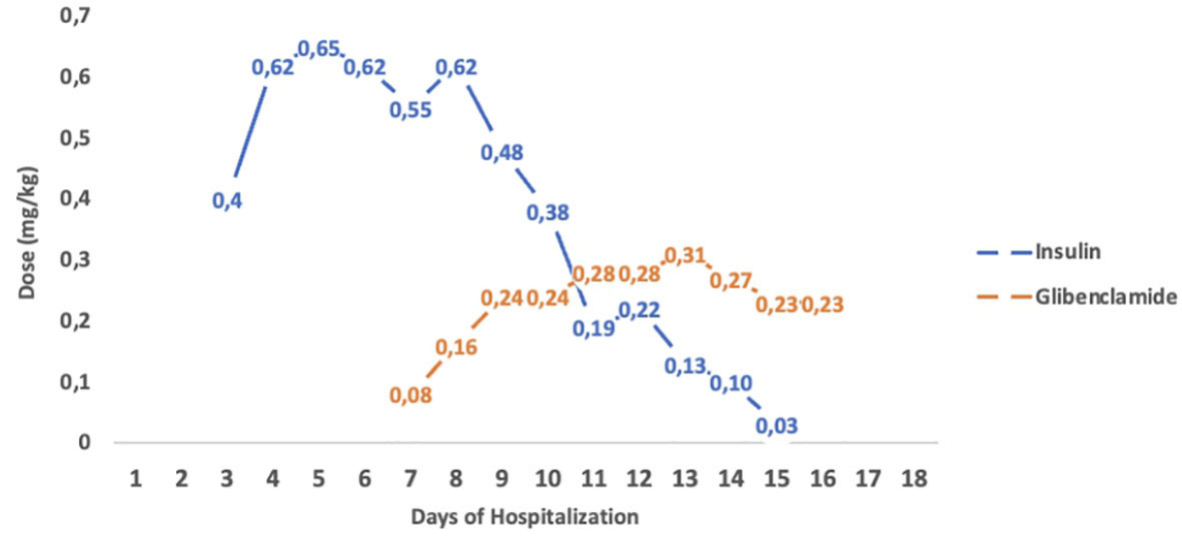
Insulin and Glibenclamide tiltration of infant 2. Modulation of insulin and glibenclamide doses (mg/kg) during hospitalization. The figure shows a progressive reduction of insulin doses during glibenclamide tiltration, till insulin was discontinued.

**Figure 3:**
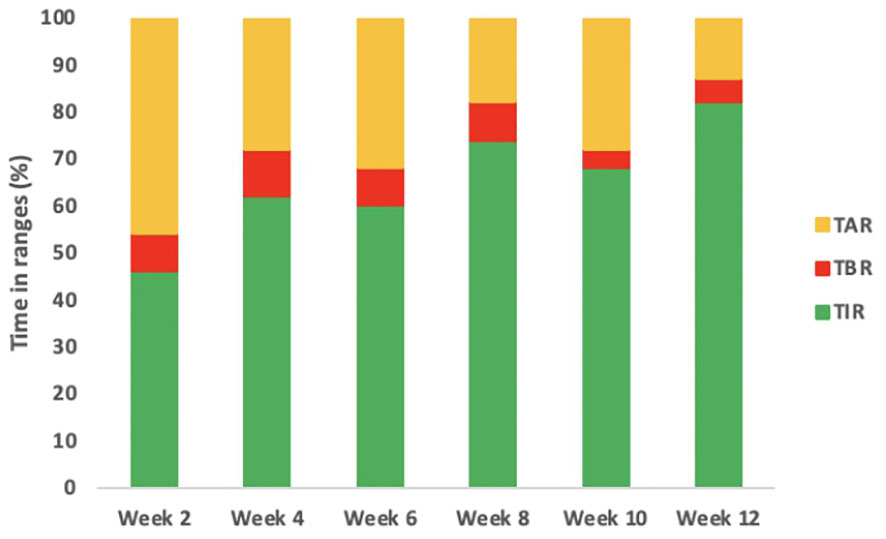
Glycemic profile of infant 2 during the follow-up. The figure describes the glycemic profile of the boy after the CGM was positioned. The timing of the follow-up is expressed in weeks from the first CGM positioning. In the first two weeks insulin therapy was gradually switched to glibenclamide therapy. In the following weeks, when the boy was only on glibenclamide treatment, an increased TIR was observed in his glucose profile. Abbreviations: CGM, continuous glucose monitoring; TIR, Time in Range.

The second case is a male infant born at 37 + 6 weeks of gestational age from non-consanguineous parents. The mother had gestational diabetes that required insulin treatment during the pregnancy. His birth weight was -2.07 DS, showing intrauterine growth retardation; breastfed, the boy presented normal weight gain in the first two months of life. He was admitted to the hospital at 51 days old for impaired general condition, severe dehydration, respiratory distress (Kussmaul), and fever. Diagnostic work-out revealed severe hyperglycemia and ketoacidosis (glucose >900 mg/dl, blood gas pH: 6.9, HCO3: 2.9 mmol/L, blood ketone 5.1 mmol/mol), confirming the diagnosis of DKA. He was transferred to the Intensive Care Unit and started treatment with intravenous fluids and regular human insulin according to ISPAD Guidelines for DKA (29). After the resolution of ketoacidosis within two days, the patient was started on CSII therapy (MiniMedTM 780 G, Medtronic, Northridge, California) and CGM (Guardian Sensor 4, Medtronic, Northridge, California), with only predictive low glucose suspend (PLGS) function on. The insulin pump was programmed as follows: two basal rates (0 IU/h or 0.025 IU/h), meal boluses (0.3 IU every 17 g of carbohydrates), and a correction factor (ISF) of 400. Short-acting insulin Lispro (Humalog, Eli Lilly, Indianapolis, IN, USA) was used. No dilution of insulin was necessary. The child was fed with maternal milk/formula milk (60 ml x 8 times/daily). Using CSII therapy, insulin administration was easier, given the small accounts of insulin needed, but glucose variability was still high (**Figure 1A**). Laboratory data detected high levels of fructosamine (427 umol/L, normal range 0-285 umol/L) and low C-peptide levels (0.07 ng/ml, normal range 0.80-4.20 ng/ml), Type 1 Diabetes (T1D) antibodies were negative (anti-insulin, anti-glutamic decarboxylase, anti-pancreatic islet cell, and anti-tyrosine phosphatase). The neurological evaluation did not show any abnormalities. A targeted NGS panel for monogenic diabetes detected the presence of a *de novo* heterozygous nonsynonymous pathogenic mutation in *KCNJ11* (c.602G>A; p.Arg201His). Thereafter, insulin administration was gradually reduced, switching simultaneously to sulfonylurea (glibenclamide) treatment in a galenic formulation. Glibenclamide was started after nine days of hospitalization with a dose of 0.08 mg/kg/day till a maximum of 0.31 mg/kg/day, divided into three oral doses per day (**Figure 1B**). Insulin therapy was discontinued 16 days after the hospital admission, seven days after glibenclamide’s first administration. **Figure 2** shows the modulation of insulin and glibenclamide doses during hospitalization. At hospital discharge, laboratory analysis showed a decreased fructosamine level, but still impaired (391 umol/L) and improved C-peptide levels (0.54 ng/ml). At home, glibenclamide treatment was modulated according to the boy’s meal requests, choosing to administer the therapy, in a smaller amount, at every meal (**Figure 1C**). The glycemic control kept improving, with no complications or side effects. At the last evaluation, after three months of treatment, the boy was 4.5 months old and showed normal psychomotor development with regular growth. Glibenclamide was administered with a dose of 0.20 mg/kg/day, divided into six meals, showing a good glycemic profile (**Figure 1D**). Laboratory data showed a normal level of fructosamine (256 umol/L) and C-peptide (1.57 ng/ml). The patient’s glycemic control during the follow-up is reported in **Figure 3**.

## Methods

### Genetic analysis

Following written informed consent, the genomic DNA of the patients was extracted from peripheral blood through the ReliaPrep Blood gDNA Miniprep System (Promega), according to the manufacturer’s recommendation. A custom-designed NGS panel including 16 genes [*ABCC8, APPL1, BLK, CEL, GCK, HNF1A, HNF1B, HNF4A, INS, KCNJ11, KLF11, LDLR, NEUROD1, PAX4, PDX1, WFS1*] involved in monogenic diabetes was used to investigate the presence of pathogenic variants in the two patients. A Kit AmpliSeq On-Demand e AmpliSeq Custom SpikeIn was utilized with a panel size corresponding to 1Mb and 41.07 kb, respectively. The sequencing probes were designed to cover all coding exons of the 16 genes and the 20 bp flanking sequence from the exon intron-exon boundary. Variant calling was performed with the Ion Reporter™ software (Thermo Fisher Scientific) and VCF files were annotated with the wANNOVAR tool. Finally post aligned, annotated, and categorized sequence data was analyzed using a personalized bioinformatics pipeline: variants were filtered by frequency, excluding those with a MAF ≥0.01 in the public databases: 1000 Genome project (https://www.internationalgenome.org/) and gnomAD (https://gnomad.broadinstitute.org/). Classification of variants was evaluated using the consensus guidelines as set out by the American College of Medical Genetics and Genomics (ACMG) guidelines (https://www.acmg.net/ACMG). Sanger sequencing was performed to confirm the presence or absence of these mutations in all the family members.

## Discussion and conclusion

We reported the different management over about 18 years of two cases of NDM due to *de novo* pathogenic variants in KCNJ11 sensitive to sulfonylurea.

The mutations detected in our patients, previously reported in the literature, have a deleterious impact on the protein structure and have been described as pathogenic for NDM (30, 31). More than 30 activating KCNJ11 variants have been related to NDM to date (19), with a spectrum of clinical manifestations of the disease, ranging from TNDM to PNDM with neurological complications (developmental delay, epilepsy, and neonatal diabetes syndrome) or maturity-onset diabetes in the young (1, 6). This variability could be explained by a mild beta cell defect caused by some mutations that may be compensated transiently but could be insufficient during puberty when is observed an increased insulin requirement (10). In our case series, the first patient has PNDM, while the second one is still too young to predict the disease’s clinical course. Their diabetes onset was similar to that seen in other children with *KCNJ11* mutations (6, 8, 10, 17). In particular, the second child showed low birth weight, a sign of intrauterine growth retardation consistent with low insulin production *in utero*, and the diabetic onset was characterized by marked hyperglycemia and ketoacidosis.

Managing infants with NDM is very difficult, given the very small insulin doses required, the lack of subcutaneous fat, and the necessity to coordinate the insulin therapy with the frequent and uncontrolled feeding schedule of the newborn, exposing the child to a high risk of hypoglycemia. Based on all these reasons, CSII is considered the best treatment option in the initial management of infants with NDM (3, 5, 10, 13, 20). However, while the first case started CSII at 9 months of age after a period of MDI therapy, the second one was switched to pump therapy directly at the onset after intravenous insulin infusion. This is due to the improved skills of pediatric diabetologists to manage neonatal diabetes thanks to technology in diabetes care like a sensor-augmented pump with integrated advanced functions able to minimize hypoglycemia episodes. CSII is also very useful for the transition from insulin to sulfonylurea, which is considered the gold standard therapy of NDM in subjects with *KCNJ11* and *ABCC8* mutations since it improves the glycemic profile but has also potential beneficial effects on neurological outcomes, especially epilepsy, muscular tone, and motor function (1, 6, 10, 21). The latest ISPAD Guidelines suggested starting the treatment as soon as children are diagnosed since the age at SU initiation may be strongly associated with the likelihood of benefits in both glycemic control and neurodevelopmental outcomes (21). Furthermore, the best improvements were seen in children who started therapy before the age of four, consistent with greater neuronal plasticity in younger individuals. Our first patient showed cognitive impairment characterized by a language delay, which was evident at four-five years of age, that initially improved after the treatment with SU was started. However, in the last years, the boy started therapy with valproic acid due to a worsening cognitive disorder, while always maintaining good glycemic control, as shown by the HbA1c levels. Our second patient is showing normal psychomotor development, even if he is still too young to predict his neurological outcomes. The positive effects of SU on the central nervous system (CNS) have been shown in previous several studies: *Bowman et al.* conducted a 10-year multicenter follow-up study of KCNJ11 permanent neonatal diabetes and reported improvement in CNS features in 47% of the patients that had CNS symptoms at the time of transition to SU therapy (32). *Dahl et al.* have demonstrated that the effect of SU on neurological outcomes eventually plateaued (3). This could be due to failure to achieve an optimal concentration of the drug in both the cerebrospinal fluid (CSF) and brain to block KATP channels, consequently affecting neuronal electrical activity (33).

Despite our first patient receiving a late genetic diagnosis of PNDM, he started an off-label treatment with SU, which has shown positive implications for his CNS. *Carmody et al.* have conducted an observational retrospective study on the Monogenic Diabetes Registry showing how there can be a delayed diagnosis of neonatal DM (34). Given the potential beneficial effect of SU treatment on the neurodevelopmental outcome and glycemic control, an empiric trial, as was performed in our first patient, is suggested when NDM is suspected.

We described here a successful treatment of KCNJ11 neonatal diabetes with SU. This was accomplished with a new modified protocol where glibenclamide was administrated before each patient’s meal, starting three times a day and up-titrated to six times a day, with the patient’s SU mean total dosage in line with the one mentioned in previous publications (3, 6). This therapeutic scheme was obtained thanks to the CGM system, which helped in more precise control of the BG levels, with continuing SU dosage adjustments in the months following the treatment start. The boy’s glycemic control kept improving during the next months, as shown by the CGM profile and by the progressive reduction of fructosamine levels, with no episodes of hypoglycemia and normal psychomotor development up to now. Our case report is the first to present a new treatment strategy for NDM with glibenclamide modulated according to the patient’s meal requests instead of the classic administration, where the medicine is given twice-three times a day (5, 31, 35).

The new diabetes technologies are changing our therapeutic approach, as shown in the case series we presented here, where we modified SU dosage very similarly to insulin treatment adjustments. As described in the literature, in the past, glibenclamide was administered two-three times a day without monitoring blood glucose levels since CGM systems were not available and patients showed a good glycemic profile, based on HbA1c levels, without developing diabetes complications. This raises the question of whether the SU dose fractionation based on the CGM profile is necessary or, due to these new technologies, we are becoming excessive in wanting to optimize the glycemic profiles of our patients with diabetes. The main limitation is that we have described only one subject with NDM treated with this new treatment strategy with glibenclamide several times daily. Our data have shown how this regimen was able to obtain more precise control of the BG levels with less hypoglycemic or hyperglycemic episodes, as shown in the CGM profiles, compared to the classical strategy with 2-3 doses but more data are yet necessary to confirm our results.

In conclusion, we have described two different therapeutic management of neonatal diabetes mellitus due to *KCNJ11* mutations, over time. In the last 20 years, many advances have led to easy molecular genetic tests thanks to NGS panels and to an improvement in hyperglycemic newborn management before, during, and after insulin weaning. A trial of oral glibenclamide must be considered in the case of *KCNJ11* variants detection since this therapy can improve neurological and neuropsychological outcomes, particularly in earlier treatment initiation. Patients treated with glibenclamide maintain good metabolic control and prevent hypoglycemia, neurological damage, and apoptosis of beta cells during long‐term administration.

## Data availability statement

The original contributions presented in the study are included in the article, and further inquiries can be directed to the corresponding author.

## Ethics statement

Ethical review and approval was not required for the study on human participants in accordance with the local legislation and institutional requirements. Written informed consent to participate in this study was provided by the participants’ legal guardian/next of kin.

## Author contributions

VM, SZ, and AM collected the anamnestic as well as the biochemical data for the patients. VM performed the literature search, reviewed, and extracted data from the papers. MG and SM performed the genetic analysis. VM performed the figures and table designing and the manuscript writing with the assistance of MG, SS, EP, FB, and IR. All authors discussed the results and contributed to the final manuscript. All authors approved the submitted version.
